# HIF-1α:CRAT:miR-144-3p axis dysregulation promotes osteoarthritis chondrocyte apoptosis and VLCFA accumulation

**DOI:** 10.18632/oncotarget.20615

**Published:** 2017-09-01

**Authors:** Jinsoo Song, Yeon-Ho Kang, Sik Yoon, Churl-Hong Chun, Eun-Jung Jin

**Affiliations:** ^1^ Department of Biological Sciences, College of Natural Sciences, Wonkwang University, Iksan, Chunbuk, Korea; ^2^ Department of Anatomy, Pusan National University School of Medicine, Yangsan, Korea; ^3^ Department of Orthopedic Surgery, Wonkwang University School of Medicine, Iksan, Chunbuk, Korea

**Keywords:** osteoarthritis, very-long-chain fatty acid, CRAT, miR-144-3p, HIF-1α, Gerotarget

## Abstract

The functional role(s) of peroxisomes in osteoarthritis remains unclear. We demonstrated that peroxisomal dysfunction in osteoarthritis is responsible for very-long-chain fatty acid (VLCFA) accumulation. Through gene-profiling analyses, we identified *CRAT* as the gene responsible for this event. *CRAT* expression was suppressed in osteoarthritis chondrocytes, and its knockdown yielded pathological osteoarthritic characteristics, including VLCFA accumulation, apoptosis, autophagic inhibition, and mitochondrial dysfunction. Subsequent miRNA profiling revealed that peroxisomal dysfunction upregulates miR-144-3p, which overlapped with the osteoarthritis pathological characteristics observed upon *CRAT* knockdown. Moreover, knocking down *HIF-1α* in normal chondrocytes suppressed *CRAT* expression while stimulating miR-144-3p. Our data indicate that deregulation of a HIF-1a:CRAT:miR-144-3p axis impairs peroxisomal function during the pathogenesis of osteoarthritis.

## INTRODUCTION

Osteoarthritis is a degenerative joint disorder characterized by cartilage extracellular matrix degradation and cell death resulting in the gradual loss of articular cartilage integrity and damage accumulation of cartilage. Chondrocytes, the only cell type present in mature cartilage maintain cartilage homeostasis by balancing catabolic and anabolic processes [1-3]. Although the primary etiology of osteoarthritis is undetermined, recent studies have provided evidence of a role in the loss of functional integrity of cellular organelles [4]. In particular, increasing evidence suggests that mitochondrial dysfunction is implicated in the pathogenesis of osteoarthritis [4, 5]. Osteoarthritis chondrocytes present decreased mitochondrial activity, mass, and depolarized mitochondrial membranes, which potentially lead to an inflammatory response *via* reactive oxygen and nitrogen species intermediates [5].

The significance of inter-organellar interaction has emerged recently [6, 7] and it is apparent that mitochondrial behavior is governed largely by interrelationships of the mitochondrion with other organelles, particularly the “peroxisome-mitochondrion connection” [6-8]. These two organelles share metabolic pathways such as those of β-oxidation of fatty acids [9-11], peroxide scavenging [12], and key fissionary components such as the fission 1 (Fis1) protein, mitochondrial fission factor protein in mammals, dynamin-related protein, and Fis1 homologue in yeast [13-16]. The functional significance of this inter-organellar relationship between mitochondria and peroxisomes in the pathogenesis of osteoarthritis has not been well-studied. We previously reported a possible interconnection between mitochondria and peroxisomes during osteoarthritis progression in diabetic patients [17]. In this study, we investigated the functional interconnection between mitochondria and peroxisomes in the pathogenesis of osteoarthritis.

Disruption of organelle integrity could affect the extra-organellar environment [18], and mitochondrial dysfunction can drive extra-organellar environmental stress, altering metabolic processes and functions in interconnected organelles. Mitochondria serve as calcium ion (Ca^2+^) sinks in eukaryotic cells [19], and mitochondrial Ca^2+^ uptake is pivotal in the regulation of cytosolic Ca^2+^ dynamics, prevention of toxic effects of high Ca^2+^ concentration, and regulation of various cellular responses including apoptosis [20]. However, verifying the signaling network linked to extra-organellar environmental modification induced by organellar dysfunction is an arduous task. Upon organelle dysfunction, signals in the extra-organellar environment might be part of a complex super-network of signals from interconnected organelles received by different cellular mechanisms that regulate diverse biological functions. Organellar dysfunctions that modulate the extra-organellar environment to trigger cellular responses through interconnected organelles remain obscure. The present study investigates extra-organellar environmental cues and responses that are triggered by cellular organelle dysfunction.

## RESULTS

### Lipid accumulation is involved in osteoarthritis pathogenesis

**Figure 1:**
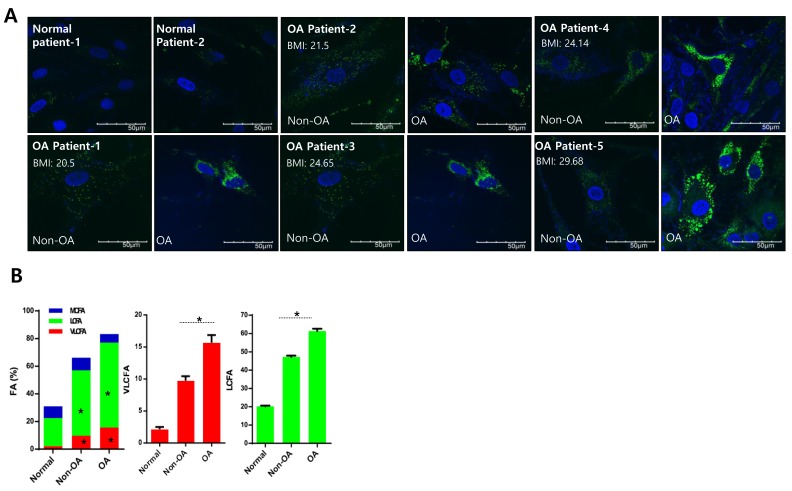
Lipid accumulation is involved in osteoarthritis pathogenesis **A**. Chondrocytes isolated from either healthy (non-osteoarthritis), or osteoarthritis cartilage from patients who underwent total knee replacement surgery and had different BMIs, were stained with BODIPY. **B.** Chondrocytes (normal, non-osteoarthritis, and osteoarthritis) were isolated, and total lipid content was analyzed using gas chromatography/mass spectrometry. Lipids were then separated into VLCFA, and either long or medium chain fatty acids (LCFA and MCFA, respectively). The data shown are representative of independent data from at least four patients.

RNA sequencing using Non-osteoarthritis chondrocyte and osteoarthritis chondrocytes isolated from healthy regions and severely damaged regions of osteoarthritis cartilage was performed and analyzed to identify the relevant signaling pathways using IPA. According to IPA, Fatty acid metabolism was one of the top potential signaling pathways of osteoarthritis pathogenesis (Data not shown). Further confirming the involvement of fatty acid metabolism in osteoarthritis pathogenesis, we analyzed altered lipid mass in normal and osteoarthritis chondrocyte cell lines stained with BODIPY (Figure 1A). Since obesity is a well-established risk factor for osteoarthritis, increased lipid accumulation might be attributed to obese osteoarthritis patients independent of pathogenesis. Cartilage samples were obtained from osteoarthritis patients with body-mass indices (BMI) ranging from 20 - 30, and chondrocytes were isolated from the healthy (non-osteoarthritis) and severely damaged (osteoarthritis) zones to investigate this (Figure 1A). Independent of BMI, we observed increased lipid accumulation in osteoarthritis chondrocytes compared to non-osteoarthritis chondrocytes of osteoarthritis patients. Lipidomics technology was applied to analyze fatty acid composition (Figure 1B). The percentage of VLCFAs significantly increased in osteoarthritis patient chondrocytes (osteoarthritis chondrocytes and non-osteoarthritis chondrocytes) compared to chondrocytes from patients without osteoarthritis. Furthermore, long-chain fatty acids (LCFAs; with aliphatic tails of 13 - 21 carbons) increased in osteoarthritis chondrocytes compared to non-osteoarthritis chondrocytes of osteoarthritis patients. These data suggest that accumulation of VLCFA might be involved in osteoarthritis pathogenesis.

**Figure 2:**
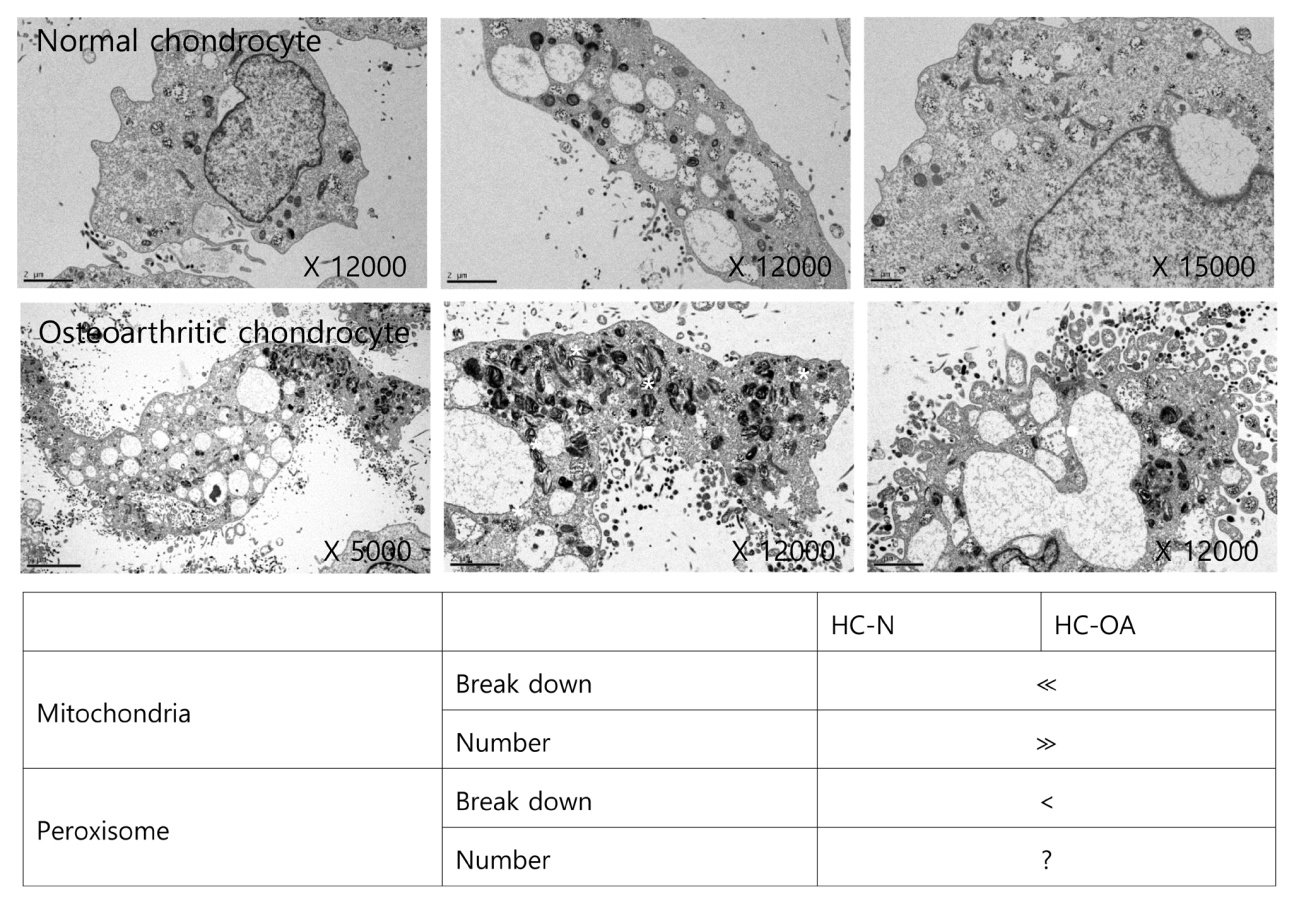
Mitochondrial and peroxisomal dysmorphology in osteoarthritis chondrocytes TEM images of human non-osteoarthritis chondrocytes and osteoarthritis chondrocytes (upper panel) were analyzed (lower panel). *indicates abnormal mitochondria and peroxisome. The data shown are representative of three independent experiments.

We analyzed TEM images to determine if the distribution of intracellular organelles in lipid metabolism differs between osteoarthritis chondrocytes and non-osteoarthritis chondrocytes, (Figure 2). We found that more mitochondria and peroxisomes were broken down in osteoarthritis chondrocytes, suggesting possible dysfunctions in lipid metabolism. Mitochondrial dysfunction has been known to occur in osteoarthritis chondrocytes [17, 21]. Since mitochondria are the sites where fatty acid molecules are broken down to generate acetyl-CoA through β-oxidation, mitochondrial dysfunction could be pivotal to osteoarthritis pathogenesis by impairing lipid metabolism. Moreover, since fatty acid chains are too long to be handled by mitochondria, VLCFAs (with aliphatic tails longer than 22 carbons; C22:0) undergo initial oxidation to octanoyl-CoA in peroxisomes. Therefore, peroxisomal dysfunction leading to the accumulation of VLCFAs might be involved in osteoarthritis pathogenesis by impairing lipid metabolism at the conjunction of mitochondria. However, the evidence to confirm the possible involvement of peroxisomal function in osteoarthritis pathogenesis is lacking.

### Downregulation of peroxisomal *CRAT* disrupts lipid homeostasis during osteoarthritis pathogenesis

**Figure 3:**
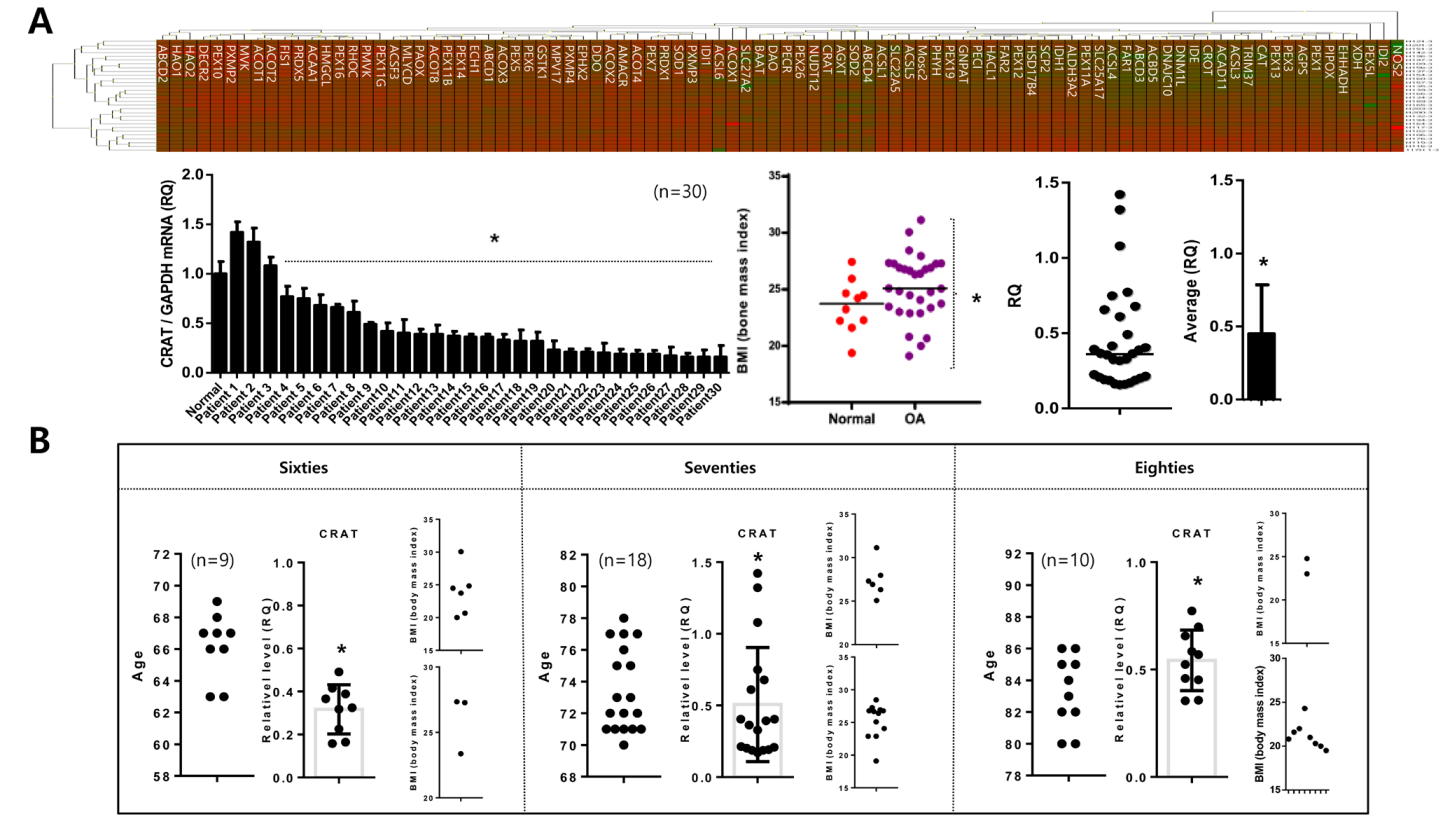
Peroxisomal gene profiling in normal and osteoarthritis chondrocytes **A**. Chondrocytes (normal and non-osteoarthritis) were isolated, and peroxisomal gene profiles were analyzed, represented as a heat map (upper panel). “Red” and “Green” indicates high and low relative expression, respectively. Similarly, the heat map for chondrocytes (normal *versus* osteoarthritis) (upper panel) is shown. Body-mass indices (BMI) of patients used in this study (left), and relative quantification of *CRAT* (expressed as fold difference compared with normal chondrocytes) are represented as dot graphs (lower panel). **B.** *CRAT* expression was analyzed by age represented as dot graph.

We analyzed the expression of 92 peroxisome-related genes (peroxogenes) between normal (*n* = 10; mean age 23.31 years) and osteoarthritis chondrocytes (*n* = 30; mean age 72.23 to identify peroxisome-related molecules responsible for pathogenic features of osteoarthritis, represented as heat maps compared to the average of normal chondrocytes (Figure 3A). *CRAT* is one of the genes whose was reduced in most osteoarthritis chondrocytes. The expression level of *CRAT* was decreased (average RQ: 0.46) in 21 of 30 patients (Figure 3A). The average BMI of normal and osteoarthritis patients in this study did not differ (23.54 and 25.21, respectively), suggesting that alteration of *CRAT* expression due to osteoarthritis pathogenesis rather than obesity. This gene is known to regulate lipid metabolism and fatty acid β-oxidation by involving in short- and medium-chain acyl-CoA transportation [22]. To investigate whether this reduction of *CRAT* in osteoarthritis chondrocytes is due to aging, we assigned osteoarthritis patients tested in this study into sixties, seventies, and eighties age groups. *CRAT* was downregulated in all ages suggesting this reduction is age-independent (Figure 3B).

**Figure 4:**
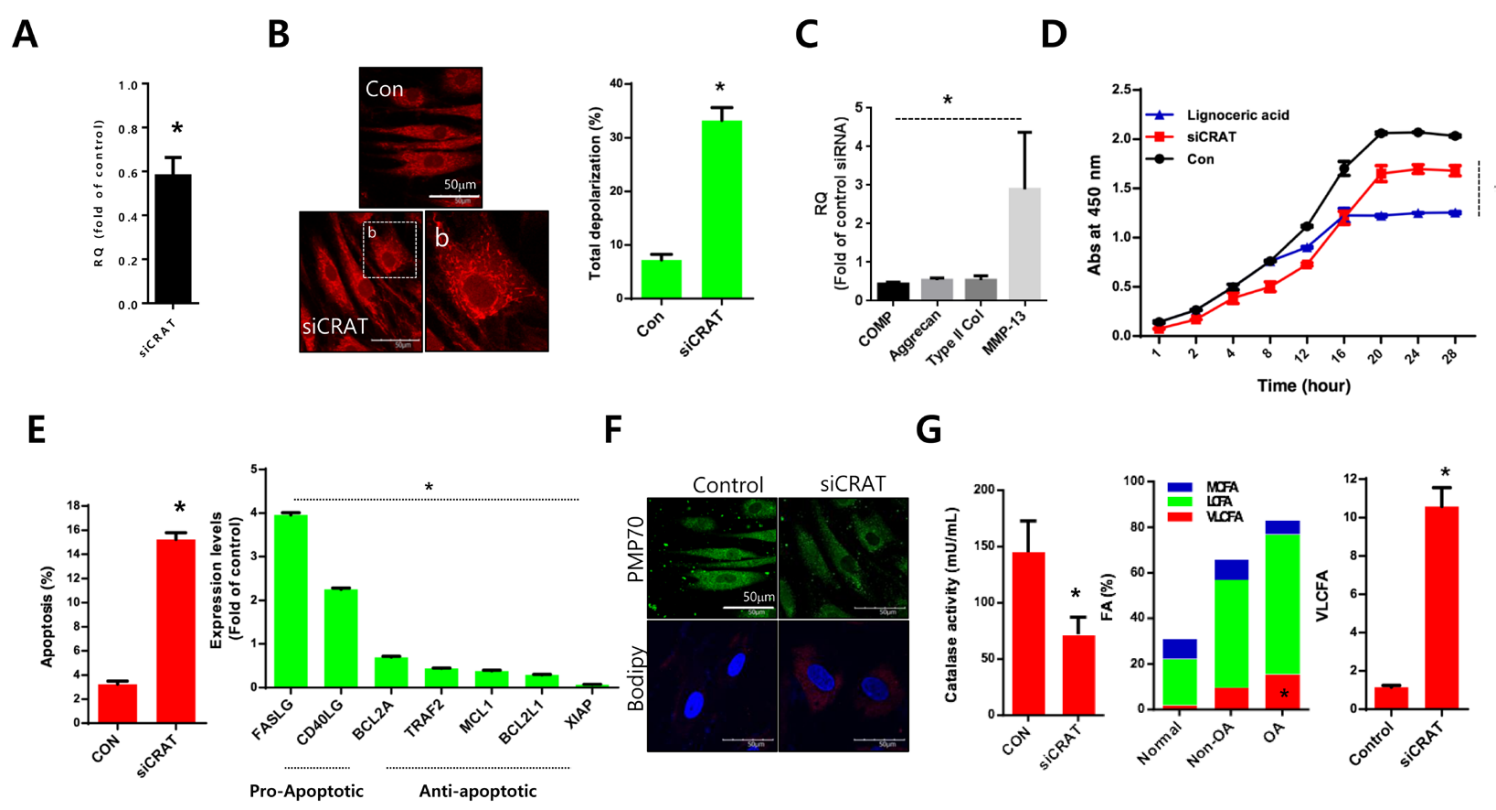
*CRAT* suppression stimulates lipid accumulation and apoptosis in human chondrocytes Normal chondrocytes were transfected with specific siRNAs against *CRAT*. **A.** The efficiency of siRNAs was analyzed by quantitative PCR (qPCR). **B.** Cells were stained with mitotracker and mitopotential was analyzed. **C.** Expression levels of *COMP*, aggrecan, and *MMP13* were analyzed by PCR. **D.** Normal chondrocytes were treated with lignoceric acid for VLCFA and cell proliferation assays. **E.** Apoptosis was analyzed using the Muse Cell Analyzer (left panel), while mRNA levels of apoptosis-related genes in *CRAT*-suppressed, chondrocytes were analyzed by PCR (right panel). **F.** Cells were stained with anti-PMP70 antibody and BODIPY. **G.** Catalase activity was analyzed (left panel). Total lipid was measured using gas chromatography/mass spectrometry and divided into VLCFA, and LCFA and MCFA (right panel). The data shown are representative of at least four individuals per experiment. Bars represent the mean ± SD of three individual experiments. *P < 0.05 *versus* control (normal).

Normal chondrocytes were transfected with specific siRNAs against *CRAT* (*siCRAT*) to determine the exact role and mechanism of CRAT in osteoarthritis pathogenesis (Figure 4A). The transfection efficiency was confirmed by quantitative (q)PCR with a 40% reduction in *CRAT* expression. With the introduction of *siCRAT*, we observed an increased number of mitochondria presenting thread-like morphology and enhanced depolarization of mitochondrial potential (Figure 4B). Downregulation of cartilage-matrix genes including *COMP*, aggrecan, and type II collagen and upregulation of the cartilage-degrading gene, matrix metallopeptidase 13 (*MMP13*) were observed in *CRAT* knockdown chondrocytes (Figure 4C). Proliferation was dramatically decreased (Figure 4D), whereas apoptosis was significantly increased (Figure 4E) in *CRAT*-knocked down chondrocytes. The expression of pro-apoptotic genes such as *FASLG* and *CD40LG* increased 4- and 2-fold, respectively. Expression of anti-apoptotic genes such as *BCL2A*, *TRAF2*, *MCL1*, and *BCL2L1* was decreased up to 2-fold upon *CRAT* knockdown in chondrocytes (Figure 4F). *CRAT* knockdown chondrocytes were strongly BODIPY-positive and displayed a 10-fold increase in the percentage of VLCFAs. However, PMP70 staining failed to show any dramatic changes (Figure 4G).

### Upregulation of miR-144-3p upon peroxisomal dysfunction stimulates cell death

**Figure 5:**
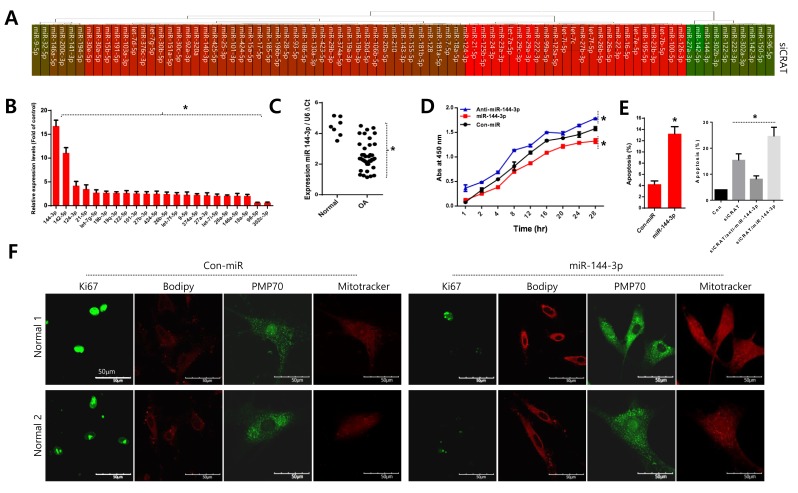
Upregulation of miR-144-3p suppresses *CRAT* in osteoarthritis pathogenesis **A**. Normal chondrocytes were transfected with specific siRNA against *CRAT* (*siCRAT*), the miRNA profile analyzed and represented as a heat map. **B.** Relative quantification of each peroxisome-related gene is expressed as fold difference compared to the control. **C.** The level of miR-144-3p was analyzed in normal and osteoarthritis chondrocytes of osteoarthritis patients. **D.** Normal chondrocytes were infected with either miR-144-3p or its inhibitor (anti-miR-144-3p), and proliferation was analyzed. **E.** Normal chondrocytes were infected with either miR-144-3p or its inhibitor (anti-miR-144-3p) in the absence or presence of *siCRAT* and apoptosis were analyzed. **F.** Cells were stained with Ki-67, PMP-70, and mitotracker. The data shown are representative of at least four individuals per experiment. Bars represent the mean ± SD of four individual experiments. *P < 0.05 *versus* control (normal).

The miRNA profile with the introduction of *siCRAT* into normal chondrocytes was analyzed to investigate if *CRAT* level affects the microenvironmental condition, and the results are represented as a heat map (Figure 5A) and a bar graph for miRNAs with a substantial change in expression (Figure 5B). Among the miRNAs altered by *CRAT* knockdown, miR-144-3p was the most upregulated (Figure 5B). Moreover, miR-144-3p was significantly upregulated in osteoarthritis chondrocytes isolated from osteoarthritis cartilage of TKR patients, compared to normal chondrocytes isolated from normal biopsy tissues (Figure 5C, right panel).

Normal chondrocytes were infected with lentiviruses expressing either miR-144-3p or its inhibitor (anti-miR-144-3p) to elucidate the role of miR-144-3p. Cell proliferation decreased in the presence of miR-144-3p and increased in the presence of anti-miR-144-3p (Figure 5D). In contrast, apoptosis increased in the presence of the miRNA-144-3p (Figure 5E, left panel). Additionally, the increased apoptosis was reverted by co-introduction of the anti-miR-144-3p construct (Figure 5E, right panel). The introduction of miR-144-3p into normal chondrocytes stimulated lipid accumulation (as assessed by BODIPY staining) and deregulated mitochondrial dynamics including increased mitochondrial fusion as revealed by long thread-like staining (Figure 5F).

### Downregulation of *HIF1α* modulates *CRAT* and miR-144-3p expression

**Figure 6:**
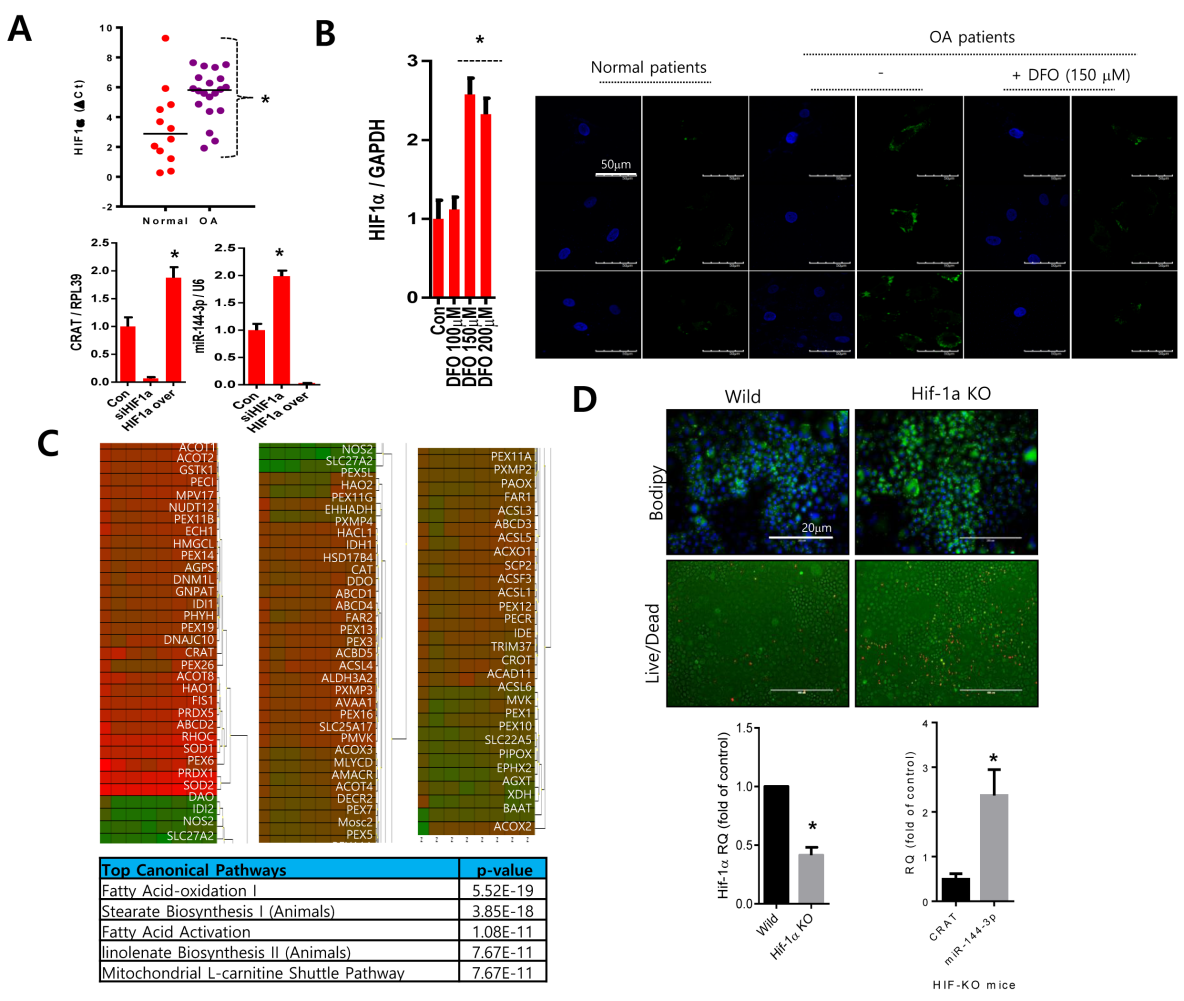
*HIF-1α* might regulate the CRAT:miR-144-3p axis **A**. The *HIF-1α* expression level was analyzed by PCR in normal or osteoarthritis chondrocytes (upper panel). Normal chondrocytes were infected with lentiviruses containing *HIF-1α-*specific siRNA (siHIF-1α) or *HIF-1α*, and the expression levels of *CRAT* and miR-144-3p were analyzed by PCR (lower panel). **B.** Osteoarthritis chondrocytes were treated with desferrioxamine (DFO) and stained with BODIPY. **C.** Peroxisomal gene profiles were analyzed and represented as a heat map (upper panel). The IPA generated top canonical pathways, and possible upstream regulators are shown (lower panel). **D.** Articular chondrocytes isolated from *HIF-1α* knock-out mice were stained with BODIPY and live and dead assay kit (upper panel) and the expression levels of *HIF-1α, CRAT,* and miR-144-3p was analyzed by PCR. Bars represent the mean ± SD of three individual experiments. *P < 0.05 *versus* control.

Since one of the main pathogenic differences in osteoarthritis is the functional inactivation and suppressed transcription of HIF-1α [23, 24], we evaluated HIF-1α participation in the regulation of *CRAT*. Consistent with previous reports [25], we observed lower levels of *HIF-1α* in osteoarthritis chondrocytes (Figure 6A upper panel). Knockdown of *HIF-1α* using its specific siRNA suppressed *CRAT* levels and stimulated miR-144-3p levels. However, over-expression of HIF-1α stimulated *CRAT* levels and suppressed miR-144-3p levels (Figure 6A lower panel). Treatment of desferrioxamine (DFO) treatment, a known HIF-1α activator, to osteoarthritis chondrocytes significantly reduced lipid accumulation (Figure 6B). We also analyzed the expression profile of peroxisome-related molecules by lentiviral transduction of *HIF-1α-*specific siRNA in normal chondrocytes which are represented in heat maps (*n* = 4; mean age 29.25 years) (Figure 6C). Ingenuity Pathway Analysis (IPA) suggested fatty acid oxidation, stearate biosynthesis, and fatty acid activation as the top canonical pathways altered by *HIF-1α.* Articular chondrocytes isolated from *HIF-1α* knock-out mice showed increases in the accumulation of lipids and chondrocyte death (Figure 6D upper panel). We observed a decrease in *CRAT* level and an increase in miR-144-3p in the articular chondrocytes of *HIF-1α* knock-out mice (Figure 6D lower panel)

## DISCUSSION

We demonstrated that peroxisomal dysfunction is involved in the pathogenesis of osteoarthritis. We observed that VLCFA accumulation in osteoarthritis chondrocytes is associated with mitochondrial dysfunction *via* downregulation of *CRAT*. The resulting upregulation of miR-144-3p stimulates apoptotic cell death. Our findings uncover an interrelationship between peroxisomes and mitochondria during osteoarthritispathogenesis.

Peroxisomes are subcellular organelles that function in multiple anabolic and catabolic processes such as β-oxidation of VLCFAs. Impaired peroxisomes have been demonstrated in several disorders including Zellweger syndrome, which is characterized by seizures, facial dysmorphism, neural heterotopia, brain dysfunction, as well as VLCFA accumulation and depletion of ether phospholipids [26, 27]. We observed abnormalities in peroxisome metabolism, including aberrant lipid metabolism during osteoarthritis pathogenesis. Peroxisomal dysfunction leads to an accumulation of VLCFAs and LCFAs during osteoarthritis pathogenesis as revealed by lipid composition in chondrocytes isolated from cartilage of osteoarthritis patients.

Once in the peroxisome, medium-chain substrates are converted to acylcarnitines by CRAT following oxidation from long-chain substrates [28-30]. In peroxisomes, CRAT is implicated in the transport of acetyl, propionyl, and medium-chain fatty acid metabolites of peroxisomal β-oxidation to the mitochondria for further metabolism, *via* a carnitine-dependent transport mechanism. We demonstrated that *CRAT* is specifically downregulated in peroxisome-related gene profiles during osteoarthritis pathogenesis. In human articular chondrocytes, knockdown of *CRAT* induced apoptosis *via* VLCFA accumulation.

MiRNAs are critical in regulatory processes that maintain homeostasis and respond to environmental changes [31] that could alter miRNA biogenesis, activities of miRNA-protein complexes, and expression of mRNA targets. The existence of miRNAs in mitochondria and their function in the translation of mitochondrial DNA-encoded mRNAs was recently demonstrated [32], which presents the possibility of mRNA regulation of organelle integrity and function. However, deregulation of organelle integrity and function could alter molecular regulation in the cytoplasm where miRNAs primarily act. We found that peroxisomal dysfunction led to upregulation of miR-144-3p.

The miR-144-3p target *ABCA1* is involved in the progression of atherosclerosis *via* the promotion of proinflammatory cytokine production in apolipoprotein E KO (*apoE*-/-) mice [33]. This gene encodes a cholesterol efflux regulatory protein that is a major regulator of cellular cholesterol and phospholipid homeostasis. We observed that upregulation of miR-144-3p, induced by peroxisomal dysfunction, stimulates apoptosis and *MMP13* induction during osteoarthritis pathogenesis. However, the biological networks of functional and regulatory pathways targeted by miR-144-3p must be investigated in detail. Our data indicate that peroxisomal dysfunction is critical to the pathology of osteoarthritis *via CRAT*-modulated accumulation of VLCFAs to modulate miR-144-3p expression as a negative signal for chondrocyte survival.

## MATERIALS AND METHODS

### Cell culture and treatments

The study was approved by the institutional review boards (Wonkwang University Ethics Committees) and performed in compliance with the Helsinki Declaration. Normal human chondrocytes were obtained as from the knee joints of adult biopsy donors with no history of joint disease. Normal (*n* = 20, average age = 56.45) or Osteoarthritic cartilage (*n* = 37, average age = 74.35) was obtained from patients diagnosed with OA which underwent joint surgery. Cartilage strips were incubated with trypsin at 37°C for 10 minutes. After removing trypsin solution, the cartilage slices were treated for 12-16 h with type IV clostridial collagenase in Dulbecco’s modified Eagle’s medium (DMEM) with 5% FCS to release cartilage cells.

### Quantitative reverse transcription PCR

Quantitative reverse transcription PCR (qRT-PCR) was performed using the StepOnePlus™ system (Applied Biosystems, Foster City, CA, USA). PCR reactions were prepared and heated to 95°C for 1 min, followed by 40 cycles of denaturation at 95°C for 10 s, annealing at the specific melting temperature for 1 min, and extension at 72°C for 1 min. Relative expression levels were normalized to the glyceraldheyde-3-phosphate dehydrogenase (*GAPDH*) housekeeping gene. The oligonucleotides used as primers were as follows: *MMP13*: 5’-ttgcagagcgctacctgagatcat-3’ antisense and 5’-tttgccagtcacctctaagccgaa-3’ sense; *COMP*: 5’- gacagtgatggcgatggtatag-3’ antisense and 5’-tcacaagcatctcccacaaa -3’ sense; *Aggrecan*: 5’-tcgaggacagcgaggcc-3’ antisense and 5’-tcgagggtgtagcgtgtagaga-3’ sense; *Type II col*: 5’-tggacgccatgaaggttttct-3’ antisense and 5’-tgggagccagattgtcatctc-3’ sense; *ADAMTS4*: 5’-cgctttgcttcactgagtagat-3’ antisense and 5’-ctgttagcaggtagcgctttag-3’ sense; *ADAMTS5*: 5’-gcactggctactatgtggtatt-3’ antisense and 5’-agccagttctcacacacttc-3’ sense; mitochondrial *CRAT*: 5’-cgggcagcgaagatgtta-3’ antisense and 5’-caaggagaagggcttcagg-3’ sense; peroxisomal *CRAT*: 5’-cttcaaggcacaccaggat-3’ antisense and 5’-actcctcctcactcacgat-3’ sense; *CROT*: 5’-gcacttcagctggcctatta-3’ antisense and 5’-actgcttcaactgtgcatga-3’ sense; *FIS1*: 5’-gacaaggccatgaagaaaga-3’ antisense and 5’-ggatttggacttggacacag-3’ sense; *CAT*: 5’-gcacttcagctggcctatta-3’ antisense and 5’ actgcttcaactgtgcatga-3’ sense; and *GAPDH*: 5’-gatcatcagcaatgcctcct-3’ antisense and 5’-tgtggtcatgagtccttcca-3’ sense.

### Cell viability and mitochondrial membrane potential assays

Cell viability and mitochondrial membrane potential were measured using the MUSE™ Cell Analyzer and the MUSE™ Mitopotential Assay Kit (EMD Millipore Corp., Billerica, MA, USA) according to the manufacturer’s instructions. The FlowJo software version 10 (http://www.flowjo.com) was used for analysis.

### Lipid staining

Cells were incubated with 1 µg/mL of BODIPY^®^542/563 (Molecular Probes, Eugene, OR, USA), washed with PBS, mounted with mounting medium (Biomedia, Foster City, CA, USA), and photographed by fluorescence microscopy using a FluoView FV1000 confocal microscope (Olympus Corp., Tokyo, Japan).

### Immunofluorescence

Cells grown on coverslips were fixed with 4% PFA in PBS for 10 min and permeabilized in PBS containing 0.1% Triton X-100 for 10 min at room temperature. Cells were incubated with anti-PMP70 (Thermo Fisher Scientific Inc., Waltham, MA, USA) or anti-Ki67 antibody (Abcam, Cambridge, MA, USA) for 1 h at room temperature and then incubated with green fluorescent protein (GFP)-conjugated goat anti-rabbit IgG (Jackson ImmunoResearch Laboratories Inc., West Grove, PA, USA) for 1 h at room temperature. Nuclei were stained with 4′,6-diamidino-2-phenylindole (Santa Cruz Biotechnologies, Santa Cruz, CA, USA).

### Immunohistochemistry

Deparaffinized tissue sections were incubated with C1,2C (Col2 3/4C_short_) antibody (IBEX Pharmaceuticals Inc., Montreal, Quebec, Canada) for 1 h at 4°C, incubated with biotinylated anti-rabbit antibody and then developed with diaminobenzidine end product of the peroxidase reaction.

### Cell proliferation assay

Cell proliferation assay was performed using Quick Cell Proliferation Colorimetric Assay Kit (Biovision, Milpitas, CA, USA) according to the manufacturer’s instructions.

### Mitochondrial staining

Cells were grown on coverslips for 3 days and stained with a 50 nM solution of MitoTracker^®^ Red CMXRos (Molecular Probes).

### Lipid analysis

Total lipids were extracted with chloroform/methanol (2:1 v/v), separated on a Sep-Pak silica cartridge column (Waters Corporation, Milford, MA, USA), and transmethylated with 0.5M sodium methoxide in methanol by heating in a sealed tube at 60 -70°C for 30 min under nitrogen. The fatty acid methyl esters were extracted with hexane. Subsequent gas chromatography-mass spectrometry analysis was performed according to standard procedures with an MAT-8430 mass spectrometer connected to an HP-5890 gas chromatograph. The latter was equipped with a DB-5 capillary column (Thermo Finnigan MAT GmbH, Bremen, Germany).

### Peroxisome-related gene profiling

The mRNAs were reverse-transcribed using the PrimeScript 1^st^ strand cDNA Synthesis Kit (Takara Bio Inc., Otsu, Japan). The relative abundance of 325 peroxisomal genes was determined by real-time PCR and expression level of the *GAPDH* gene (PCR primers: 5′-gatcatcagcaatgcctcct-3′ antisense and 5′-tgtggtcatgagtccttcca-3′ sense) was used as a reference. Analysis and visualization of gene expression data were carried out using the GenEx software (bioMCC, Freising-Weihenstephan, Germany).

### Cell transfection

Cells were transfected with siRNAs against *CRAT* (*siCRAT*) (Bioneer, Korea) using the Lipofectamine 3000 Transfection Reagent (Invitrogen, Carlsbad, CA, USA) and incubated at 37°C for 48 h.

### Apoptotic cell death assay

Cells were incubated at room temperature for 5 min in the dark with the Annexin V & Cell Death Assay Kit (EMD Millipore Corp.). The fluorescence levels of annexin V were analyzed using a MUSE™ Cell Analyzer (EMD Millipore Corp.).

### Cell death profiling

Cell death profiling was performed using the miScript PCR System (QIAGEN GmbH, Hilden, Germany), which includes miRNA-specific primers for the analysis of pathways related to apoptosis, autophagy, and necrosis (Cell Death PathwayFinder RT^2^ Profiler™ PCR Array; QIAGEN).

### Experimental osteoarthritis mouse model

Experimental osteoarthritis was induced by DMM surgery in 8 week-old male wild-type and catalase KO mice. Mock-operated animals injected with empty lentiviruses (mock transduction) were used as controls for the DMM mice. Histological and biochemical analyses were performed either 8 weeks post-DMM surgery with every 2 weeks intra-articular injection of 1×10^9^ plaque-forming units of lentiviruses. Cartilage destruction in mice was examined using Safranin O staining.

### miRNA profiling

RT^2^ miRNA PCR arrays (QIAGEN) were analyzed using the StepOnePlus™ system (Applied Biosystems), and miRNA expression data were normalized to the expression levels of the *RNU6* snRNA. Analysis and visualization of gene expression data were carried out using the GenEx software (bioMCC).

### miRNA PCR assay

TaqMan Small RNA Assay was used using a miR144-3p-specific primer (Applied Biosystems). Real-time PCR was carried out on the StepOnePlus™ system (Applied Biosystems) at 95°C for 10 min, followed by 40 cycles of heating at 95°C for 15 sec and 60°C for 1 min.

### Production of lentiviral particles

Lentiviruses containing miR144-3p precursor (miR144-3p), miR144-3p specific siRNA (anti-miR-144-3p) and negative control lentivirus (Applied Biological Materials Inc., Richmond, BC, Canada) were packaged using the 3rd Generation Packaging Mix (Applied Biological Materials) and transduced into the human 293FT host cell line using Lentifectin™ (Applied Biological Materials) in Opti-MEM^®^ I medium (Invitrogen). The supernatant was collected, and lentiviral particles were concentrated using the Lenti-X Concentrator (Clontech, Mountain View, CA, USA).

### Generation of Col2-Cre directed conditional Hif1a Knockout mice

Hif1a floxed mice and Col2CreERT mice were obtained from Jackson Laboratories (Bar Harbor, ME). Hif1a^flox/flox^ mice were bred to Col2CreERT (Mackem et al., 2006) to obtain Col2CreERT+; Hif1a^flox/flox^ (Hif1a CKO) mice. Littermate Col2CreERT; Hif1a^flox/WT^ mice were used as a control. The genotype of Col2CreERT and Hif1a alleles were analyzed by PCR as previously described. The primers for Col2CreERT were sense: 5’-CACTGCGGGCTCTACTTCAT-3’ and anti-sense: 5’-ACCAGCAGCACTTTTGGAAG-3’. The primers for Hif1a were sense: 5’-TGCATGTGTATGGGTGTTTTG-3’ and anti-sense: 5’-GAAAACTGTCTGTAACTTCATTTCC-3’. Genomic tail DNA was amplified by PCR.

### Primary culture of immature articular chondrocyte

Immature articular chondrocytes were isolated from the femoral heads, femoral condyles and tibial plateau of 5∼6 days old mice. Briefly, 1 litter of 5∼6 days old mice were euthanized using CO_2_ overdose followed by thoracotomy. The femoral heads, femoral condyles, and tibial plateau were removed from the long bones, and translucent cartilage was cut into small pieces, placed in 1 X PBS, and then rinsed. Chondrocytes were isolated by digestion using collagenase D (Roche) and seeded at a density of 8 × 10^3^ cells/cm^2^ in culture medium consisting of DMEM (Sigma, cat. No. D5546) supplemented with 2 mM L-Gln, 10% fetal bovine serum (FBS, Gibco-Invitrogen), 50 U/ml penicillin, and 50 µg/ml streptomycin (Gibco-Invitrogen) at 37^°^C in a humidified atmosphere of 5% CO2. After 5 days of amplification, the cells were incubated in the presence or absence of 1 µM of 4-hydroxytamoxifen (4-OHT) for 24 hours.

### Transmission electron microscopy

Cultured chondrocytes fixed in Karnovsky’s solution were post-fixed with 1% OsO4, contrasted with 2% uranyl acetate, and embedded with Epoxy Embedding Medium Kit (Sigma-Aldrich, St. Louis, MO, USA). Epoxy embedded block was ultra-microsectioned using EM UC7 (Leica, Vienna, Austria). Microstructure image was analyzed with 120 kV transmission electron microscope (HITACHI #H-7650, Japan).

### Statistical analysis

A two-tailed Student’s *t*-test or one-way ANOVA followed by the Student-Newman-Keuls post hoc test was used to determine the significance of the differences between results, with *p* < 0.05 taken as indicating a significant difference.
