# Correction: HIF-1α:CRAT:miR-144-3p axis dysregulation promotes osteoarthritis chondrocyte apoptosis and VLCFA accumulation

**DOI:** 10.18632/oncotarget.27091

**Published:** 2019-07-16

**Authors:** Jinsoo Song, Yeon-Ho Kang, Sik Yoon, Churl-Hong Chun, Eun-Jung Jin

**Affiliations:** ^1^ Department of Biological Sciences, College of Natural Sciences, Wonkwang University, Iksan, Chunbuk, Korea; ^2^ Department of Anatomy, Pusan National University School of Medicine, Yangsan, Korea; ^3^ Department of Orthopedic Surgery, Wonkwang University School of Medicine, Iksan, Chunbuk, Korea

**This article has been corrected:** During the assembly of Figure 1A, the same image was inadvertently used for OA patient 1 and OA patient 3. In addition, a duplicate copy of the figure 1B bar graph was accidently copied into Figure 4G. The proper Figure 1A and 4G are shown below. The authors declare that these corrections do not change the results or conclusions of this paper.

Original article: Oncotarget. 2017; 8:69351–69361. 69351-69361 . https://doi.org/10.18632/oncotarget.14540

**Figure 1:**
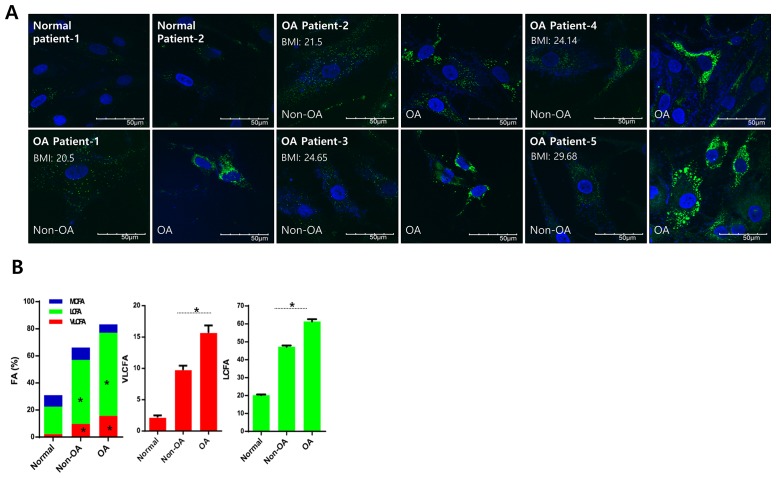
Lipid accumulation is involved in osteoarthritis pathogenesis. **A.** Chondrocytes isolated from either healthy (non-osteoarthritis), or osteoarthritis cartilage from patients who underwent total knee replacement surgery and had different BMIs, were stained with BODIPY. **B.** Chondrocytes (normal, non-osteoarthritis, and osteoarthritis) were isolated, and total lipid content was analyzed using gas chromatography/mass spectrometry. Lipids were then separated into VLCFA, and either long or medium chain fatty acids (LCFA and MCFA, respectively). The data shown are representative of independent data from at least four patients.

**Figure 4:**
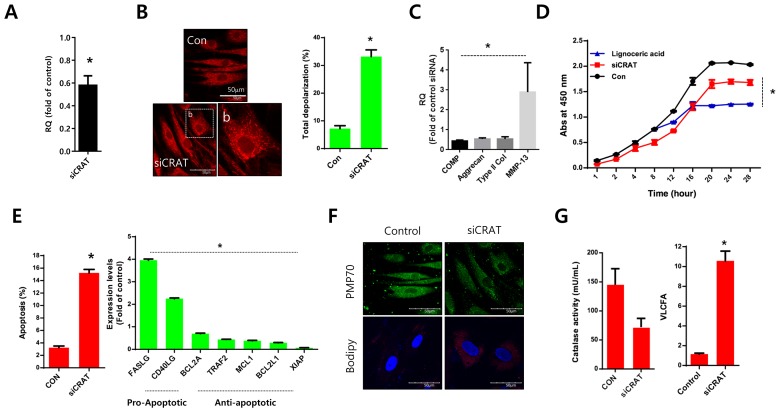
*CRAT* suppression stimulates lipid accumulation and apoptosis in human chondrocytes. Normal chondrocytes were transfected with specific siRNAs against *CRAT*. **A.** The efficiency of siRNAs was analyzed by quantitative PCR (qPCR). **B.** Cells were stained with mitotracker and mitopotential was analyzed. **C.** Expression levels of *COMP*, aggrecan, and *MMP13* were analyzed by PCR. **D.** Normal chondrocytes were treated with lignoceric acid for VLCFA and cell proliferation assays. **E.** Apoptosis was analyzed using the Muse Cell Analyzer (left panel), while mRNA levels of apoptosis-related genes in *CRAT*-suppressed, chondrocytes were analyzed by PCR (right panel). **F.** Cells were stained with anti-PMP70 antibody and BODIPY. **G.** Catalase activity was analyzed (left panel). VLCFA was measured using gas chromatography/mass spectrometry. The data shown are representative of at least four individuals per experiment. Bars represent the mean ± SD of three individual experiments. *P < 0.05 *versus* control (normal).

